# Elevated Dietary Vitamin D Supplementation During Winter can Mimic Vitamin D Levels Induced by Summer Daylight Exposure in Atlantic Salmon

**DOI:** 10.1155/anu/3152332

**Published:** 2026-06-09

**Authors:** Frederike Keitel-Gröner, David Lausten Knudsen, Eirik Hoel, Margunn Sandstad, Kjetil Berge

**Affiliations:** ^1^ Skretting AS, Stavanger, Norway; ^2^ Skretting Aquaculture Innovation, Stavanger, Norway; ^3^ FishLab AS, Stavanger, Norway

**Keywords:** aquaculture, cholecalciferol, *Salmo salar*, tissue deposition, vitamin D metabolism

## Abstract

Vitamin D_3_ (cholecalciferol) is an essential nutrient for vertebrate calcium homeostasis, skeletal integrity, and immune function. In aquaculture, declining use of fishmeal and fish oil has reduced dietary vitamin D_3_ levels in salmon feeds, raising questions about tissue deposition and nutritional quality of farmed fish. This study aimed to investigate the metabolic fate of elevated dietary vitamin D_3_ in farmed Atlantic salmon (*Salmo salar*) under commercial production conditions in southern Norway. Fish were fed either a control diet (~4000 IU/kg vitamin D_3_) or a test diet with a 10‐fold higher inclusion (~40,000 IU/kg vitamin D_3_) for 32 weeks from November to July 2025. Plasma, liver, and muscle samples were analyzed for vitamin D_3_ and its metabolites [25(OH)D_3_ and 1,25(OH)_2_D_3_], alongside plasma biomarkers of health. Salmon tolerated the high dietary vitamin D_3_ without observed adverse effects on growth and mortality. Elevated dietary inclusion significantly increased vitamin D_3_ concentrations in plasma, liver, and muscle, with less pronounced effects on hydroxylated metabolites, compensating for lower levels during winter months. However, seasonal variation was evident in all tissues investigated. Overall, the results demonstrate that high dietary vitamin D_3_ supplementation increases tissue concentrations in Atlantic salmon without compromising health, thereby improving the nutritional profile of farmed salmon fillets for human consumption.

**Summary**

• High dietary vitamin D_3_ (10× standard level) was well tolerated with no adverse effects on growth and mortality.
• Vitamin D_3_ supplementation markedly increased tissue concentrations in plasma, liver, and muscle, while hydroxylated metabolites increased to a lesser extent, helping compensate for naturally lower winter levels.
• Seasonal variation strongly influenced vitamin D_3_ status.
• The nutritional quality of salmon fillet improved as elevated dietary vitamin D_3_ enhanced tissue deposition.

## 1. Introduction

Vitamin D refers to a group of fat‐soluble secosteroids essential for calcium and phosphorus homeostasis, skeletal integrity, and immune functions in vertebrates [1, 2]. In humans, vitamin D deficiency is a widespread public health concern, particularly in regions with limited sunlight exposure. Insufficient levels have been linked to osteoporosis, a disease characterized by demineralization of bones with an increased risk of fractures, increased susceptibility to infections, and a range of diseases including cardiovascular diseases, diabetes, cancer, depression, and multiple sclerosis [2]. Endogenous synthesis in the skin from 7‐dehydrocholesterol via UV‐B radiation (290–315 nm) is the primary source for vitamin D [3], but in view of the modern Western lifestyle, dietary intake remains crucial [4]. Fish and fish products are among the richest natural dietary sources of vitamin D [4], with concentrations ranging from 6 to 33 µg/100 g of fresh salmon [2].

In humans, vitamin D_3_ (cholecalciferol), the form found in animal‐derived foods, undergoes hydroxylation in the liver to form 25‐hydroxyvitamin D_3_ [25(OH)D_3_] [5], the main circulating metabolite [6]. A second hydroxylation step in the kidneys produces 1,25‐dihydroxyvitamin D_3_ [1,25(OH)_2_D_3_], the biologically active form that interacts with the vitamin D receptor [1]. At the circulatory level, vitamin D attaches to vitamin D binding protein and is stored in adipose tissue [7]. Serum level of 25(OH)D_3_ above 30 ng/mL (75 nmol/L) is considered adequate for human health [8].

In aquaculture, the nutritional composition of the fish feed directly influences the vitamin D content of the farmed fish. Atlantic salmon (*Salmo salar*), a key species in global aquaculture, is thought to rely primarily on dietary sources of vitamin D [9]. Historically, fishmeal and fish oil provided sufficient vitamin D_3_, but the increasing use of plant‐based feed ingredients has led to a decline in feed vitamin D content. Hannisdal et al. [10] reported that vitamin D levels in both feed and fillets of Norwegian farmed Atlantic salmon declined from 2006 to 2021. The feed content dropped from 0.68 to 0.10 µg/g and fillet levels from 0.091 to 0.067 µg/g. Consequently, a 150 g salmon portion provided 91% of the recommended vitamin D intake in 2006 but only 76% in recent years, according to EFSA recommendations (15 µg/day) [11].

In response to these trends, the European Union revised its regulation on vitamin D_3_ inclusion in fish feed (Regulation (EU) 2019/849), increasing the maximum allowed level from 3000 to 60,000 IU/kg (corresponding to 0.075–1.5 µg/g) for salmonids. Although safety assessments indicated that increased feed levels would not exceed tolerable upper intake levels for human consumers, uncertainties remained regarding the transfer efficiency of vitamin D_3_ from feed to fish flesh.

Previous studies have demonstrated that elevated dietary vitamin D can increase fillet concentrations [12, 13], and environmental factors such as light exposure may also influence circulating levels of 25(OH)D_3_ [14]. It was recently shown that salmon reared in land‐based aquaculture facilities without natural light exposure had low levels of vitamin D_3_ in the muscle [15]. Furthermore, Fossen et al. [16] exposed Atlantic salmon fingerlings indoors to UV‐B light and found significantly higher vitamin D_3_ concentrations in the muscle after 4 and 10 weeks of exposure compared to a control group. Finally, Husebø et al. [17] found a seasonal variation in plasma levels of vitamin D_3_, 25(OH)D_3_, and 1,25(OH)_2_D_3_, suggesting endogenous synthesis of vitamin D_3_ and metabolites under natural light conditions.

This study investigates the effects of a high vitamin D_3_ diet on the distribution and concentration of vitamin D metabolites in plasma, liver, and fillet of commercially farmed Atlantic salmon. By quantifying these metabolites, we aimed to elucidate the metabolic fate of dietary vitamin D_3_ and assess its potential to enhance the nutritional profile of farmed salmon. The findings have implications for both fish physiology, feed formulation, and public health nutrition.

## 2. Materials and Methods

### 2.1. Experimental Design and Diets

The trial was conducted at Hestholmen (Rogaland, Norway), a commercial site (site number 14136) operated by Grieg Seafood Rogaland AS under the R&D licenses RS0001 and RS0002, which were provided by the Norwegian Directorate of Fisheries to Skretting AS. Hestholmen is located 59.058° N, 5.445867° E. Daylength during the trial period ranged from 6 h 11 min (December 21, 2024) to 18 h 29 min (June 21, 2025), with a daily average of 12 h 20 min.

From November 2024 (week 44–48), between 122 362 and 155 897 Atlantic salmon post‐smolts with a pen average weight of 935 ± 57 g (range from 785 to 1069 g) were transferred into eight net pens on‐site (Aqualine, SCALE AQ, Norway and Polarcirkel, AKVA group, Norway; 160 m circumference, 45 m deep nets). From stocking until 20. On June 2025, a lighting regime with LED lights was installed at 10 m depth in all pens (3 × 1000 W). All pens were fed the same commercial diet (Skretting AS, Norway) until the feeding trial started. Experimental diets were fed for about 32 weeks (week 48/2024 to week 28/2025) to four pens in each of the two groups. Thereafter, a commercial diet (Skretting AS, Norway) was provided to all pens until the harvest. The experimental diets consisted of a standard grower diet (Control) and a test diet based on a commercially available skin health diet (Armor, Skretting AS) with the inclusion of vitamin D (cholecalciferol [D_3_], Trouw Nutrition Nederland BV, The Netherlands) at 40,000 IU/kg (1 µg/g). Feeds were produced in three series, varying in composition to adapt to the increasing size of the fish with 600‐feed (7 mm) for fish from 600 to 1200 g, 1200‐feed (9 mm) for fish from 1200 to 2500 g, and 2500‐feed (9 mm) for fish above 2500 g (Table 1). Fish were fed with 600‐feed for the first 4–6 weeks, followed by 1200‐feed for another 10–12 weeks, before fish were fed 2500‐feed until the end of the experimental feeding period. All feeds were produced under commercial conditions at the Skretting Stavanger plant on a commercial extruder. Vitamin D was added as a dry powder to the meal mix before extrusion. The proximate composition of the diets is shown in Table 2. Diets were fed by Grieg Seafood Rogaland according to their standard procedures.

**Table 1:** Composition of experimental diets (g/100 g of dry matter).

Raw materials	Control 600	Control 1200	Control 2500	Test 600	Test 1200	Test 2500
Fish meal	7.91	5.22	7.79	7.86	5.31	7.84
Krill meal	0.37	0.00	0.00	2.49	0.00	0.00
Wheat gluten	19.00	12.29	11.18	16.31	13.33	7.38
Wheat	9.08	7.24	6.01	8.33	5.66	7.70
Soy protein concentrate	17.51	21.19	8.60	23.20	23.23	22.83
Horse beans	3.90	6.32	9.10	3.93	8.96	8.62
Guar meal	9.71	12.05	10.38	3.15	3.12	3.13
Pea protein concentrate	0.00	0.83	8.24	0.00	3.26	3.20
Sunflower meal	1.01	0.82	0.77	0.00	0.62	3.12
Fish oil (South American)	7.98	9.94	11.65	11.99	12.85	12.42
Fish oil (Scandinavian)	0.92	0.00	0.00	1.70	1.07	0.30
Rapeseed oil	16.12	19.50	22.36	12.48	15.59	16.97
Lecithin	1.00	1.01	1.03	1.01	1.01	1.19
Vitamin mix	0.19	0.20	0.20	0.42	0.42	0.42
Vitamin D^a^	0.00	0.00	0.00	0.81	0.79	0.78
Mineral mix	2.04	1.62	1.24	1.99	1.73	1.36
Pigment	0.05	0.05	0.05	0.05	0.05	0.05
Other	3.18	1.72	1.40	4.28	3.01	2.69
Nutritional values						
DE (Mj/kg)	20.9	21.7	22.3	20.4	21.2	21.6
DP (g/kg)	358	326	293	358	326	293
Vitamin D sources (%)						
From fishmeal/fishoil	64	65	68	10	10	11
From vitamin mix	36	35	32	6	6	6
From vitamin D^a^	0	0	0	84	84	83

*Note:* Values for raw materials were obtained from actual productions of feed during the whole trial period, whereas nutritional values are theoretical mean values.

^a^Vitamin D supplementation.

**Table 2:** Proximate composition (g/100 g sample) and vitamin D content (IU/kg) of experimental diets of three different sizes (600: 7 mm pellet intended for fish 600–1200 g; 1200: 9 mm pellet intended for fish 1200–2500 g; 2500: 9 mm pellet intended for fish >2500 g).

Proximate composition	600		1200		2500	
	Control	Test	Control	Test	Control	Test
Ash	4.7	5.4	4.2	4.9	4.4	5.0
Fat	29.2	28.7	32.8	33.0	37.8	36.3
Moisture	7.7	7.6	7.1	6.9	6.8	6.6
Protein	42.4	42.5	37.9	38.0	34.5	35.0
Vitamin D	n.a.	44,900	4340	35,350	n.a.	35,000

Abbreviation: n.a., not analyzed.

Seawater temperatures for the trial period ranged from 4.3°C (week 8/2025) to 14.6°C (week 28/2025), with an average of 8.5 ± 2.7°C for the trial period.

### 2.2. Fish Sampling

**Figure 1:**
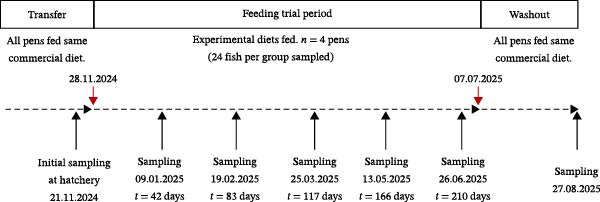
Experimental set‐up with key events and sampling dates. Red arrows mark experimental diets feeding period.

The initial biological sampling took place at the hatchery 1 week before the feeding trial commenced at the end of November (28.11.2024). During the experimental feeding phase, five additional samplings were carried out between January and June 2025, specifically in January (*t* = 42 days), February (*t* = 83 days), March (*t* = 117 days), May (*t* = 166 days), and June (*t* = 210 days). After the experimental feeding period concluded, a final sampling was conducted in August, 51 days later (Figure 1 and Table 3).

**Table 3:** Seawater parameters at samplings.

Water parameters	09.01.2025	19.02.2025	25.03.2025	13.05.2025	26.06.2025	27.08.2025
Temperature (°C)	7.6	5.4	6	8.6	13.4	13.7
Oxygen (%)	96	101	99	88	95	96
Salinity (ppm)	34	32	31	33	33	30

Fish were collected from the net pens using a sweep net by on‐site personnel. A total of six fish from each cage were randomly sampled. Fish were euthanized in an overdose of Finquel Vet. (MSD Animal Health, Norway) before handling. Fish were weighed (nearest g) and fork length (nearest 1/2 cm) was measured. Blood was sampled from the caudal vein using 4 mL lithium‐heparinized vacuum tubes (Greiner Bio‐One, Austria) according to Keitel‐Gröner et al. [18]. Tubes were gently inverted several times to allow mixing of blood and anticoagulant, before plasma was separated on site by centrifugation at 2500 × *g* for 6 min (Compact Star CS4 Microfuge, VWR, USA). Pooled samples were prepared by transferring 200 µL plasma from each fish into a 2 mL polypropylene microcentrifuge tube.

Additionally, pooled samples of the liver and left‐side Norwegian Quality Cut (NQC) muscle were taken. The NQC is a standardized cross section of the fish extending from posterior of the dorsal fin to the anterior of the anal fin [19].

Plasma samples were stored at −80°C and liver and muscle samples were stored at −20°C prior to further processing.

### 2.3. Sample Processing

Plasma samples (500 µL) were analyzed in Skretting AI Laboratory (Stavanger, Norway) using an Indiko Clinical Chemistry Analyzer (Thermo Fisher Scientific, USA) using commercially available kits for albumin, alanine aminotransferase (ALAT), aspartate aminotransferase (ASAT), calcium, chloride, creatine kinase (CK), creatinine, lactate dehydrogenase (LDH), magnesium, phosphorus, potassium, sodium, total protein, and urea. All kits were purchased from Thermo Fisher Scientific (USA).

Additionally, plasma samples were analyzed for vitamin D_3_, 25(OH)D_3_, and 1,25(OH)_2_D_3_ levels by FishLab AS (Stavanger, Norway) according to Husebø et al. [17]. Homogenized pooled liver and muscle samples were also analyzed for vitamin D_3_ and 25(OH)D_3_ by FishLab AS (Stavanger, Norway) following the procedure outlined for whole‐body homogenate in Husebø et al. [17].

### 2.4. Feed Samples and Analysis

Proximate composition of feed samples was analyzed by the Skretting AI laboratory (Stavanger, Norway). Dietary ash and moisture contents were determined gravimetrically after heating to 555°C for 17 h (ash) or drying at 97–105°C for 16–18 h (moisture). Samples were processed and analyzed according to internal methods at the ISO 17025 accredited laboratory based on NMKL method number 23, third edition, 1991. These adapted methods have been validated by repeated ring testing with external laboratories. Dietary fat content was measured using near magnetic resonance imaging (NMR) according to NMKL method number 199, 2014. Nitrogen contents were analyzed by Kjeldahl, according to NMKL method number 6, fourth edition (2003) and multiplied by 6.25 to estimate the dietary crude protein content. Vitamin D_3_ levels in feed were analyzed using HPLC (EN 12821:2009 mod.) by Eurofins (Klepp Stasjon, Norway) during routine quality checks.

### 2.5. Calculations

Specific Growth Rate (SGR) (%/d) was calculated using the formula below, considering days of starvation for the calculation of number of days in the trial.

SGR=Final weight/initial weight1/number of days−1 ×100.

### 2.6. Statistics

Pens were considered the experimental units. All statistical analyses were performed using JMP 18.1.1 (JMP Statistical Discovery, LLC). Data were tested for normality [20] and homogeneity of variances [21]. Differences between the two groups were assessed using either an independent samples *t*‐test for normally distributed data or the Mann Whitney test for nonparametric data [22]. For vitamin D metabolites, a two‐way ANOVA (Fit Least Squares) was additionally performed with group, month, and the interaction term group × month included as model effects to evaluate main effects of both group differences and potential seasonal effects (shown in the Supporting Information) and interactions between the two following general recommendations by Quinn and Keough [23]. Statistical significance was defined as *p* < 0.05.

### 2.7. Ethics Statement

All handling of fish complied with the Guidelines of EU legislation (Directive 2010/63/EU) and Norwegian legislation (LOV‐2009‐06‐19‐97).

## 3. Results

### 3.1. Growth and Mortality

During the experimental diet feeding period, growth parameters did not differ significantly between groups. The average SGR was 0.62 ± 0.05 in the Control group and 0.60 ± 0.05 in the Test group. Mortality was higher in the Control group (8.6 ± 5.0%) compared to the Test group (6.8 ± 2.7%).

### 3.2. Vitamin D in Feed

Vitamin D concentration in the control feed (1200) was measured at 4340 IU/kg and at 44,900, 35,350, and 35,000 IU/kg in the Test 600‐, 1200‐, and 2500‐feeds (Table 2), respectively. This corresponds to a deviation of +3.2% from the target in the Control feed and deviations of +12.3%, –11.7%, and –12.6% in the respective Test feeds.

### 3.3. Plasma Parameters

Only a few plasma parameters showed statistically significant differences between groups (Table 4). In June, ALAT and ASAT levels were significantly higher in the Control group. Additionally, urea levels were elevated in the Test group in June.

**Table 4:** Plasma parameters (mean and standard deviation) in Control and Test group at various timepoints throughout the trial.

Plasma parameters	Group	Control				Test			
	Month	November	March	June	August	November	March	June	August
Albumin (g/L)	Mean	18.2	16.8	16.6	16.5	18.2	16.5	16.8	16.7
	Std Dev	1.0	0.9	0.5	0.8	1.7	1.6	1.0	0.4
ALAT (U/L)	Mean	49.6	39.9	118.0 ^∗^	25.4	51.1	53.1	81.5	27.9
	Std Dev	8.7	13.4	16.4	2.3	18.0	17.0	15.7	7.9
ASAT (U/L)	Mean	755	408	895 ^∗^	623	680	418	553	513
	Std Dev	54	91	135	123	63	65	152	134
Calcium (mmol/L)	Mean	3.63	3.74	3.45	3.20	3.64	3.47	3.48	3.13
	Std Dev	0.46	0.25	0.07	0.18	0.63	0.24	0.11	0.06
Chloride (mmol/L)	Mean	126	146	150	147	126	142	148	141
	Std Dev	11	8	4	9	24	3	3	6
CK (U/L)	Mean	13,832	7643	31,984	11,870	9588	8805	17,876	8905
	Std Dev	1976	5593	7935	7333	3025	5087	11,714	4875
Creatinine (µmol/L)	Mean	24.80	8.55	2.98	3.53	27.70	10.10	4.84	2.40
	Std Dev	3.95	1.96	1.91	2.35	3.31	0.63	3.10	1.56
LDH (U/L)	Mean	1128	657	557	348	917	541	505	494
	Std Dev	205	255	174	102	588	125	52	36
Magnesium (mmol/L)	Mean	1.37	1.16	2.07	1.89	1.38	1.20	2.09	1.64
	Std Dev	0.25	0.27	0.12	0.06	0.06	0.31	0.10	0.12
Phosphorous (mmol/L)	Mean	5.96	7.24	6.83	5.51	6.27	6.90	7.50	6.06
	Std Dev	0.80	0.52	0.43	0.30	1.23	1.38	0.45	0.34
Potassium (mmol/L)	Mean	7.30	7.81	5.59	4.43	7.29	7.40	6.40	4.60
	Std Dev	1.01	1.21	0.98	0.57	1.82	1.51	0.62	0.10
Sodium (mmol/L)	Mean	164	174	170	168	163	168	169	163
	Std Dev	14	8	3	7	29	5	2	5
Total protein (g/L)	Mean	47.3	39.8	38.0	38.7	45.4	41.4	38.7	38.0
	Std Dev	3.7	3.0	1.4	2.5	4.8	6.1	2.0	0.0
Urea (mmol/L)	Mean	2.04	1.65	1.62	1.33	2.17	1.97	1.94 ^∗^	1.23
	Std Dev	0.23	0.31	0.10	0.06	0.34	0.53	0.16	0.15

*Note:* Statistically significant differences between groups at a given timepoint are indicated with an asterisk ( ^∗^).

### 3.4. Vitamin D Metabolites in Plasma

**Figure 2:**
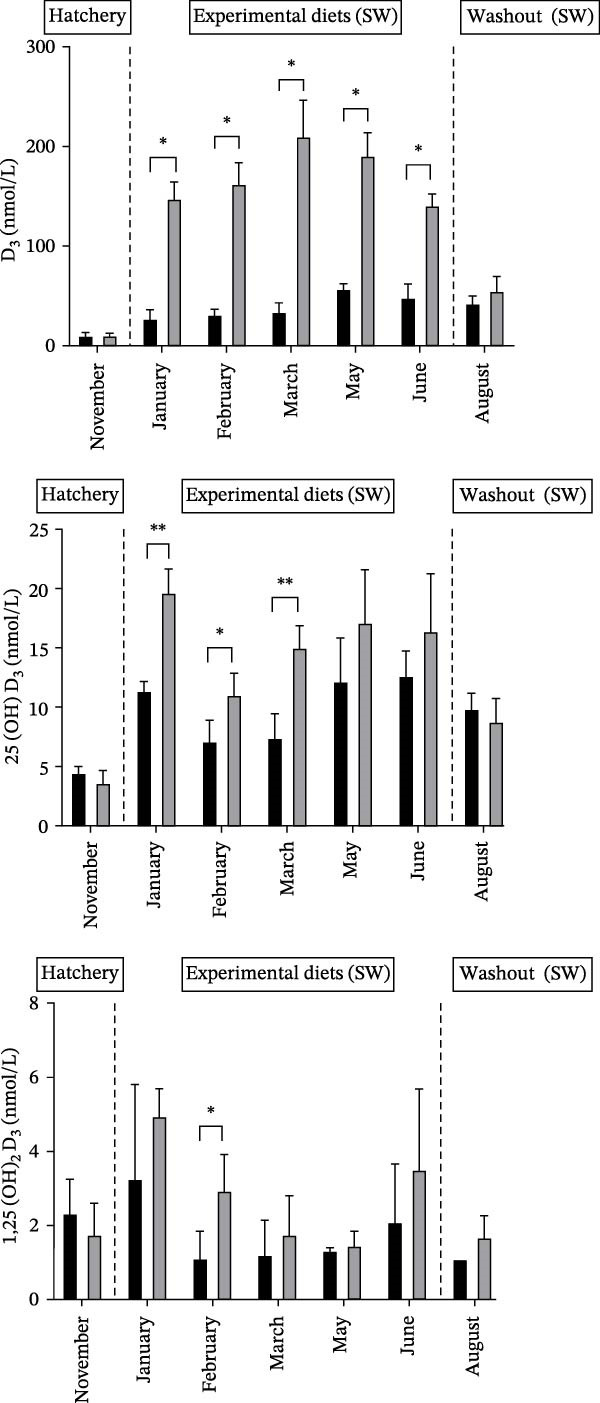
Plasma concentrations of vitamin D metabolites (nmol/L) in the Control (black bars) and Test (gray bars) group at different time points during the trial. Dotted lines indicate the experimental diet period in seawater (SW). Bars represent mean and standard deviation (*n* = 3–4 pooled samples). Asterisks indicate statistically significant differences between groups ( ^∗^ *p* < 0.05,  ^∗∗^ *p* < 0.01).

No significant differences in any vitamin D metabolites were observed in samples collected indoors in November (Figure 2), with both groups showing similar vitamin D_3_ levels (around 10.5 ± 2.2 nmol/L). In the Control group, vitamin D_3_ increased moderately, reaching 57.7 ± 4.7 nmol/L in May, before declining slightly to 42.8 ± 7.3 nmol/L in August. The Test group exhibited statistically significant higher vitamin D_3_ levels compared to the Control group from January to June, peaking in March at 210.5 ± 35.8 nmol/L, which corresponds to a sixfold higher level compared to the Control group.

Group differences in hydroxylated metabolites (25(OH)D_3_ and 1,25(OH)_2_D_3_) were generally less pronounced. However, 25(OH)D_3_ levels were consistently higher in the Test group during the experimental feeding period (January to June), with statistically significant differences observed from January to March. During this time, levels in the Test group were up to twice as high as those in the Control group. In contrast, 1,25(OH)_2_D_3_ did not exhibit a clear pattern in relation to group differences or seasonal variation. Notably, in February, the Test group showed a 2.6‐fold higher concentration compared to the Control group, which was statistically significant. Both metabolites were unexpectedly elevated in January across groups.

### 3.5. Vitamin D Metabolites in Liver and Muscle

**Figure 3:**
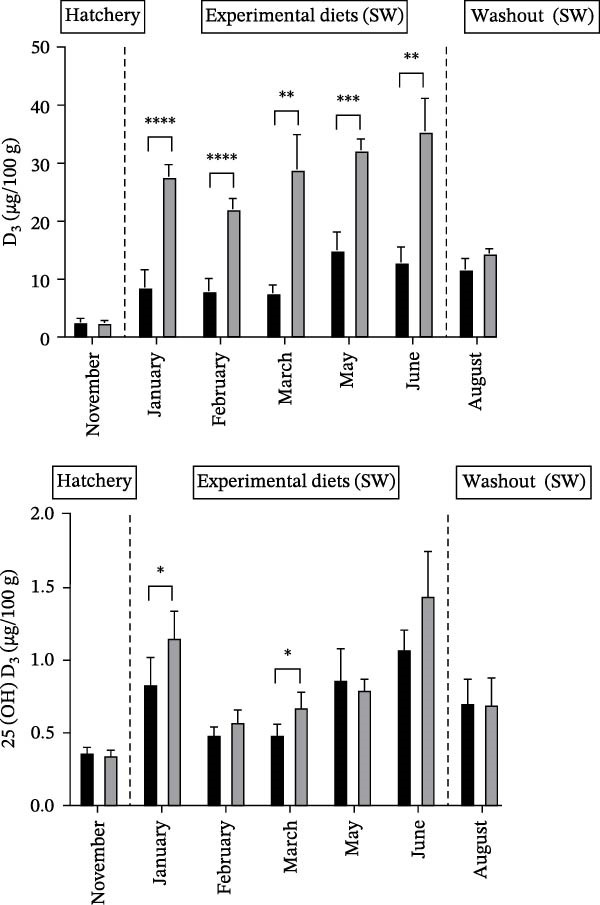
Liver concentrations of vitamin D metabolites (µg/100 g) in the Control (black bars) and Test (gray bars) group at different time points during the trial. Dotted lines indicate the experimental diet period in seawater (SW). Bars represent mean and standard deviation (*n* = 3–4 pooled samples). Asterisks indicate statistically significant differences between groups ( ^∗^ *p* < 0.05,  ^∗∗^ *p* < 0.01,  ^∗∗∗^ *p* < 0.001,  ^∗∗∗∗^ *p* < 0.0001).

In liver tissue (Figure 3), vitamin D_3_ concentrations were significantly higher in the Test group compared to the Control group from January to June, with up to a four‐fold increase observed in March. Both groups started at similar levels in November (Control: 2.6 ± 0.7 µg/100 g; Test: 2.5 ± 0.5 µg/100 g). The Control group showed a seasonal trend, with peak concentrations in May and June, although these increases were not statistically significant. The Test group followed a similar seasonal pattern, but with consistently higher levels, reaching a peak in June (35.5 ± 5.7 µg/100 g), which was more than double the Control group’s peak in May (15.0 ± 3.1 g/100 g). By August, after a period of 51 days with similar vitamin D_3_ levels in the feeds, levels were comparable again (Control: 11.7 ± 1.9 µg/100 g and Test: 14.5 ± 0.8 µg/100 g).

The metabolite 25(OH)D_3_ (Figure 3) tended to be higher in the Test group, with statistically significant differences in January and March, reaching up to 1.4‐fold higher concentrations in the liver. Aside from elevated levels in January, a seasonal increase was also evident in May and June. However, the rise in D_3_ concentrations in the Test group was not mirrored by a proportional increase in 25(OH)D_3_. Overall, 25(OH)D_3_ concentrations were substantially lower than those of D_3_ in both groups.

**Figure 4:**
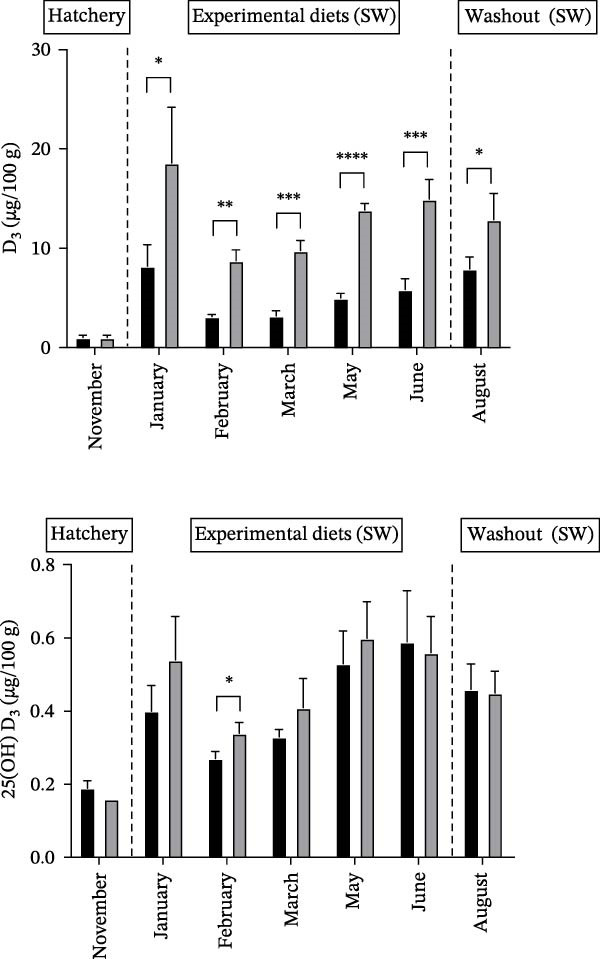
Muscle concentrations of vitamin D metabolites (µg/100 g) in the Control (black bars) and Test (gray bars) group at different time points during the trial. Dotted lines indicate the experimental diet period in seawater (SW). Bars represent mean and standard deviation (*n* = 3–4 pooled samples). Asterisks indicate statistically significant differences between groups ( ^∗^ *p* < 0.05,  ^∗∗^ *p* < 0.01,  ^∗∗∗^ *p* < 0.001,  ^∗∗∗∗^ *p* < 0.0001).

In muscle tissue (Figure 4), vitamin D_3_ and 25(OH)D_3_ concentrations followed a similar pattern to what was observed in the liver tissue, although overall levels were lower. There were no statistically significant differences between the groups in November. At all other sampling points, the Test group showed significantly higher levels, including August. Muscle tissue displayed the clearest seasonal effects. Except for January, both groups had statistically significant increases compared to November. In the Control group, levels rose from 1.0 ± 0.2 µg/100 g in November to 7.9 ± 1.2 µg/100 g in August, which was statistically significant. In the Test group, May and June showed the highest concentrations, significantly higher than in the winter months (except for January).

For 25(OH)D_3_, no statistically significant differences were found between groups in the muscle tissue, except for February. However, a clear seasonal trend was observed, with significantly higher concentrations in May and June in both groups (again, except for January).

## 4. Discussion

This study documents the metabolic fate of substantially elevated dietary vitamin D_3_ and its metabolites in the plasma, liver, and muscle tissue of Atlantic salmon under commercial production conditions in southern Norway. The NRC does not specify a vitamin D_3_ requirement for Atlantic salmon [24], but previous research has suggested a dietary requirement of 0.06 µg/g (2400 IU/kg) and tissue saturation occurring at 0.08 µg/g (3200 IU/kg) for post‐smolt salmon [25]. Here, fish in the Control group were fed with a commercial diet (~4000 IU/kg vitamin D_3_, 0.1 µg/g), while fish in the Test group received a diet with an approximate 10‐fold increase of vitamin D_3_ aiming at 40,000 IU/kg (1 µg/g) from the end of November to the beginning of July 2025. No adverse effects on growth, mortality, and selected plasma biomarkers were observed at the tested dose. Horvli et al. [12] conducted one of the few controlled studies on the effects of very high dietary vitamin D_3_ on Atlantic salmon. The authors evaluated three dietary concentrations (0.04, 2.21, and 28.68 µg/g diet, which are equivalent to 1600, 88400, and 1,147,200 IU/kg, respectively) over a period of 11 weeks. The highest dose was about 29 times greater than in the Test feed used in the current study. The authors did not find any significant differences in weight gain, feed conversion, or mortality between groups either. Fish had an initial weight of around 177 g in the study by Horvli et al. [12], compared to 935 g in the study presented here. Furthermore, Flo et al. [15] observed no effect on mortality from their test diets but reported that Atlantic salmon showed higher SGR when vitamin D_3_ increased from 0.2 to 1.12 µg/g (8000 to 44,800 IU/kg) with constant phosphorus. In this indoor experiment, fish initially weighed 453 g and were fed the diets for 17 weeks. In a recent review on vitamin and trace mineral requirements in Atlantic salmon [26], the authors pointed out an important interaction between vitamin D and K, previously documented in humans and mammalian model organisms, playing a key role in bone and cardiovascular health. The review also emphasized the importance of adequate dietary calcium and phosphorus to prevent bone mineral loss. In Atlantic salmon, Graff et al. [27] investigated these interactions, but the levels of vitamin D and K in the diets were not low enough to produce deficiency symptoms, and no notable effects were seen. In Gilthead Seabream (*Sparus aurata*) larvae, various combinations of vitamin D_3_ and vitamin K_3_ demonstrated a clear interaction between those two essential nutrients, affecting bone development and calcium homeostasis [28]. Therefore, future studies on dietary vitamin D fortification should also take vitamin K into account.

### 4.1. Plasma Biomarker

High dietary vitamin D_3_ did not negatively impact the plasma biomarkers measured here. Plasma calcium concentrations remained comparable between groups throughout the trial, indicating that calcium metabolism was not affected by the high dietary vitamin D3. Nevertheless, to fully assess long‐term effects on calcium balance, analysis of whole‐body or bone mineral content would be necessary. Graff et al. [27] demonstrated that Atlantic salmon fry fed diets containing 0.2, 5, or 57 µg/g vitamin D_3_ for 14 weeks showed no differences in kidney or whole body calcium content, concluding that calcium metabolism in early‐feeding fry was resistant to short‐term exposure to very high dietary vitamin D3.

Urea concentrations were significantly higher in the Test group in June, a biomarker that can reflect impaired kidney function or dehydration. Since vitamin D_3_ influences calcium and phosphate metabolism, which can affect renal function, it is possible that the increased dietary vitamin D_3_ contributed to the increase in urea. Both calcium and magnesium are excreted via the kidneys, and sustained elevated plasma levels could lead to oversaturation and possibly to calcium deposits within the kidney [29]. However, as the observed urea values remained within the normal range previously established for farmed Atlantic salmon [18], the increase may reflect a physiological adaptation to higher vitamin D_3_ intake rather than a pathology. Further studies are needed to determine if elevated urea is directly linked to vitamin D_3_ supplementation or to other environmental and physiological factors.

### 4.2. Plasma Vitamin D Metabolites and Seasonal Influence

A 10‐fold increase in dietary vitamin D_3_ did not result in proportional rises across plasma, liver, or muscle, but significantly elevated concentrations were observed in all tissues.

Plasma D_3_ levels in the Control group were comparable to values reported by Husebø et al. [17] for a location in the same production area, confirming a strong seasonal influence. In the Test group, plasma D_3_ increased six‐fold compared to Control, peaking at 210 nmol/L, which falls within the range (up to 300 nmol/L) reported across 12 Norwegian salmon farms [17]. Additionally, elevating dietary intake of vitamin D during the winter season produced plasma concentrations of vitamin D and its metabolites that were comparable to or exceeded those observed in the Control group during summer.

Consistent with earlier literature, vitamin D_3_ remained the dominant metabolite in salmon plasma, unlike humans, where 25(OH)D_3_ is the primary circulating form [30]. Group differences in 25(OH)D_3_ were modest but generally higher in the Test group, while 1,25(OH)_2_D_3_ showed minimal variation. Concentrations of 1–5 nmol/L observed here align with ranges reported by Husebø et al. [17] (1–10 nmol/L), Eide Graff et al. [31] (0.5–1.5 nmol/L), and Lock et al. [32] (0.5–4.2 nmol/L). The transient peak in January may reflect seawater transfer, a process previously linked to elevated 1,25(OH)_2_D_3_ during adaptation in salmonids [32, 33].

### 4.3. Vitamin D Deposition in Liver and Muscle

Vitamin D_3_ concentrations were consistently higher in the liver than in the muscle tissue, reflecting the liver’s role as the primary storage organ [12]. From a human nutritional perspective; however, muscle tissue is more relevant, and the observed increases in fillet vitamin D_3_ content indicate that dietary fortification can enhance the nutritional value of farmed salmon, albeit to a limited extent. In the Test group, muscle concentrations did not exceed 20 µg/100 g, and fold‐changes compared to the Control group remained below three. This contrasts with Horvli et al. [12], who reported tissue concentrations rising proportionally to feed inclusion levels. In comparison, Hannisdal et al. [10] documented a median fillet concentration of 0.076 µg/g (0.054–0.100 µg/g) in Norwegian farmed Atlantic salmon, which aligns closely with the Control group values observed in the present study.

Furthermore, data from this study suggest that vitamin D_3_ can accumulate in the liver over time, indicated by a slight increase in vitamin D_3_ measurements from January to June. Graff et al. [27] found a trend of decreasing percentage of retained vitamin D_3_ with increasing levels in the feed and ascribed this finding to a possible mechanism of protecting the fish from vitamin D_3_ intoxication. Furthermore, the results indicate that 51 days following the end of the experimental feeding period, vitamin D_3_ levels in liver tissue returned to levels observed in the Control group. Conversely, vitamin D_3_ levels in the muscle tissue of the Test group remained significantly higher than those in the Control group.

Therefore, vitamin D_3_ storage and metabolism seem to be differently regulated in different tissues and to be a more dynamic process in the liver tissue compared to muscle tissue. In humans, it was shown that vitamin D bound in the adipose tissue was not readily available but present in an esterified form. Individuals suffering from vitamin D deficiency with low levels of circulating vitamin D in plasma could have large amounts of vitamin D stored in the adipose tissue. This form of ester‐bound vitamin D could re‐enter into circulation after heavy exercise or weight loss [34]. Our study also shows that vitamin D appears to be stored more permanently in muscle, possibly due to ester bonds [35], whereas in the liver, vitamin D levels returned to normal within 2 months.

While vitamin D_3_ levels were significantly elevated in the present study, the hydroxylated metabolite 25(OH)D_3_ showed only modest increases and fewer statistically significant differences between groups. This is in line with observations by Rider et al. [36] 2025, who found only limited in vivo conversion of dietary vitamin D_3_ to 25(OH)D_3_ in trout. This suggests that not all dietary vitamin D_3_ is metabolized into 25(OH)D_3_, raising questions about the bioavailability and physiological demand for hydroxylated forms under a high dietary intake. It is possible that excess vitamin D_3_ is stored rather than converted, particularly in the liver, which is known to be the primary storage site [12, 37], but also in muscle tissue. Furthermore, Graff et al. [27] speculated that the low and quite stable levels of 25(OH)D_3_ in fish and marine mammals protect the animals from vitamin D_3_ intoxication. The authors suggested that when plasma levels of 25(OH)D_3_ only increase slightly in response to elevated dietary vitamin D_3_, the amount of 1,25(OH)_2_D_3_ available to interact with target cells will also be limited. As a result, toxicity is unlikely to occur, which may help to explain why fish and marine mammals appear to tolerate high dietary levels of vitamin D3.

Vitamin D_3_ and 25(OH)D_3_ seem to follow a seasonal pattern with peak concentrations in May and June in the muscle and liver tissue. This is in line with observations previously reported by Fjelldal et al. [14] and Fossen et al. [16]. Recent findings also indicate that endogenous vitamin D_3_ production via photochemical pathways was undetectable in trout raised indoors, further confirming that vitamin D_3_ synthesis depends on exposure to light [36].

It should be noted that the analytical CV for vitamin D_3_ and 25(OH)D_3_ was about 12%. Consequently, part of the observed variation between months may also be attributable to the analytical uncertainty. Overall, the results emphasize the importance of continued investigation into the ways light exposure and photoperiod influence vitamin D metabolism in salmon, as well as their vitamin D needs when facing challenging environmental conditions.

### 4.4. Implications for Feed Formulation and Public Health

The results support the EU’s decision to raise the maximum allowed vitamin D_3_ inclusion in salmonid feed (Regulation (EU) 2019/849). Given the decline in natural vitamin D_3_ content in commercial salmon feeds due to plant‐based feed ingredients [10], strategic fortification can help maintain salmon as a valuable dietary source of vitamin D for humans. Strategic dietary fortification of vitamin D is particularly important for fish raised indoors and for those kept in net pens during seasons with limited sunlight exposure. In these environments, fish have reduced opportunities for endogenous vitamin D synthesis due to the absence or scarcity of natural UV light, making them more reliant on dietary sources to achieve adequate tissue concentrations.

### 4.5. Study Limitations

In addition to differences in vitamin D concentrations, the diets varied in their raw material composition, which may influence growth performance, nutrient utilization, mortality, and the absorption of fat‐soluble vitamins. These factors should be considered when interpreting the results as they restrict the attribution of observed effects (or absence of effects) exclusively to vitamin D fortification. Furthermore, only two levels of vitamin D were evaluated, and a more frequent sampling schedule during the washout period would have provided a greater insight into the dynamics of vitamin D depletion. Additionally, incorporating other indicators, such as bone mineralization and vertebral integrity, would have further substantiated the safety assessment.

## 5. Conclusions

Here, under full‐scale commercial conditions, we could demonstrate that winter dietary fortification can achieve tissue vitamin D levels comparable to those observed during summer, ensuring that farmed salmon remains a robust source of vitamin D for human consumption throughout the year.

## Author Contributions

Frederike Keitel‐Gröner, Eirik Hoel, Margunn Sandstad, and Kjetil Berge conceived the study. Frederike Keitel‐Gröner, Eirik Hoel, and Kjetil Berge performed the experiment and collected the field samples. David Lausten Knudsen developed and performed the analytical methods for vitamin D analysis. Frederike Keitel‐Gröner and Kjetil Berge performed the statistical analysis and all authors contributed to the interpretation of the results. Frederike Keitel‐Gröner wrote the first draft of the manuscript and all authors contributed to reviewing and editing.

## Funding

This study was partly funded by the Research Council of Norway (Grant 328674).

## Disclosure

Skretting is a fish feed company and FishLab offers vitamin D analyses on fish.

## Conflicts of Interest

The authors declare no conflicts of interest.

## Supporting Information

Additional supporting information can be found online in the Supporting Information section.

## Supporting information


**Supporting Information** Figures (S1–S3) display statistical differences between groups and months, highlighting seasonal variations in Vitamin D metabolites.

## Data Availability

Data supporting the findings of this study are available from the corresponding author upon reasonable request.
