# Supplementary material for: Elevated Dietary Vitamin D Supplementation During Winter can Mimic Vitamin D Levels Induced by Summer Daylight Exposure in Atlantic Salmon
Source: Aquac Nutr. 2026 Jun 9;2026:3152332. doi: 10.1155/anu/3152332 (PMC13248107; doi:10.1155/anu/3152332)
Supplement: Supplementary file 1 — Supporting Information Figures (S1–S3) display statistical differences between groups and months, highlighting seasonal variations in Vitamin D metabolites. [file ANU-2026-3152332-s001.docx]

**Supplementary material**

Plasma

|  |
| --- |
|  |
|  |

Figure S1. Plasma concentrations of vitamin D metabolites (nmol/L) in the Control (black bars) and Test (grey bars) groups at different time points during the trial. Bars represent mean and standard deviation (n = 3–4 pooled samples). Different upper-case letters indicate statistically significant differences between groups and months, illustrating both group differences as well as seasonal variations.

Liver

|  |
| --- |
|  |

Figure S2. Liver concentrations of vitamin D metabolites (µg/100 g) in the Control (black bars) and Test (grey bars) groups at different time points during the trial. Bars represent mean and standard deviation (n = 3–4 pooled samples). Different upper-case letters indicate statistically significant differences between groups and months, illustrating both group differences as well as seasonal variations.

Muscle

|  |
| --- |
|  |

Figure S3. Muscle concentrations of vitamin D metabolites (µg/100 g) in the Control (black bars) and Test (grey bars) groups at different time points during the trial. Bars represent mean and standard deviation (n = 3–4 pooled samples). Different upper-case letters indicate statistically significant differences between groups and months, illustrating both group differences as well as seasonal variations.
